# The Influence of In Vitro Gastrointestinal Digestion on the Chemical Composition and Antioxidant and Enzyme Inhibitory Capacities of Carob Liqueurs Obtained with Different Elaboration Techniques

**DOI:** 10.3390/antiox8110563

**Published:** 2019-11-16

**Authors:** Raquel Rodríguez-Solana, Natacha Coelho, Antonio Santos-Rufo, Sandra Gonçalves, Efrén Pérez-Santín, Anabela Romano

**Affiliations:** 1 Faculdade de Ciências e Tecnologia (MeditBio), Universidade do Algarve, Campus de Gambelas, 8005-139 Faro, Portugal; nrcoelho@ualg.pt (N.C.); smgoncalves@ualg.pt (S.G.); 2 Laboratory of Plant Pathology, Area of Crop Protection, Andalusian Institute of Agricultural Research and Training (IFAPA), Centro ‘Alameda del Obispo’, Apartado 3092, 14080 Cordoba, Spain; antonio.santos.rufo@juntadeandalucia.es; 3 Graduate School of Engineering and Technology, Universidad Internacional de La Rioja (UNIR), Av. de la Paz, 137, Logroño, 26006 La Rioja, Spain; efren.perez@unir.net

**Keywords:** antioxidant capacity, *Ceratonia siliqua* L., enzyme inhibition, gallic acid, gastrointestinal digestion, total phenolic content, total flavonoid content

## Abstract

Carob liqueur is a traditional Mediterranean alcoholic beverage obtained via a wide range of production techniques contributing to the different organoleptic attributes of the final product. The aim of this research was to evaluate the stability of the chemical composition and biological capacities (antioxidant and enzyme inhibition) under in vitro simulated gastrointestinal digestion of liqueurs prepared by flavouring the fig spirit with carob pulp by maceration, distillation, percolation, or aqueous and hydro-alcoholic infusions. For this purpose, the phenolic and furanic compositions, the total phenolic (TPC) and flavonoid (TFC) contents, antioxidant capacity (AC), and enzyme inhibitory potential against acethylcholinesterase, tyrosinase, α-glucosidase and α-amylase enzymes were evaluated. The content of gallic acid decreased after gastrointestinal digestion, while TPC, TFC, and AC significantly increased after each digestion phase. Overall, no significantly different enzyme inhibitions (*p* < 0.05) were observed among digested liqueurs, with moderate inhibition against acethylcholinesterase and tyrosinase (enzymes related with neurodegenerative diseases), and potent and low inhibitory capacities for α-glucosidase and α-amylase, respectively (ideal conditions employed in antidiabetic therapy). The study indicates that hydro-alcoholic infusion and maceration were the most appropriate methods to obtain liqueurs with higher values of the aforementioned parameters and safe levels of toxic furanics.

## 1. Introduction

Carob liqueur is a traditional alcoholic beverage produced in countries of the Mediterranean basin using carob pulp. This pulp is extracted from the carobs, the fruits of carob tree (*Ceratonia siliqua* L., Fabaceae), one of the most useful Mediterranean trees [1]. Previous works emphasize the beneficial contribution to human health of compounds from carob pulp, including various nutrients (such as several important minerals and vitamins) [2], as well as phenolic compounds with great antioxidant capacity (AC) [3,4,5,6], enzyme inhibitory potential [7], and antiproliferative effect [8], among others.

The chemical composition of carob liqueur depends on different features such as the type and strength of the plant-based alcohol, the *C. siliqua* cultivar, and the different processing steps performed on carob pulp (particle size, roasting, etc.) [9]. However, there are also other aspects of the liqueur preparation that can influence its final characteristics, namely the extraction method and conditions. Different carob pulp/plant-based alcohol ratios, the temperature of extraction, the homogeneity of the plant mass and the duration of extraction are key factors for obtaining high plant extraction yields [9,10].

Traditionally, different extraction techniques have been applied by alcoholic beverage industries in order to obtain plant extracts as base ingredients for liqueurs. Maceration, percolation, infusion and distillation are traditional extraction procedures [11]. The use of a specific method depends on the importance of highlighting specific attributes during the liqueur manufacturing process. Maceration, percolation and infusion methods are based on diffusion and osmosis phenomena [12]. Infusion and maceration are quite similar techniques, both involving the steeping of sliced/crushed fruits in a solvent. Infusion is a method not often applied at an industrial scale, while maceration is the oldest method employed in the beverage industry and is still used today. In the infusion method, extraction, generally in water, occurs at a high temperature, and thus the plant material can incur some damage, such as a loss of flavour compounds. However, the high temperature can favour the extraction of certain nonthermolabile compounds. In maceration, the proper alcohol concentration for extraction must be high and agitation is required for the diffusion of compounds in order to avoid the equilibrium of extracted substances. This technique is recommended for extracting soluble and thermolabile active substances. Percolation is a process whereby the solvent circulates from the bottom to the top of the tank, through a container holding the plant mass from which the flavour is extracted over and over [13,14]. Finally, distillation employs thermal treatment with water and alcohol to allow the recovery of uncoloured hydro-alcoholic extracts, formed by intensely aromatic volatile compounds, used afterwards in liqueurs. In this process it is common to blend alcohol and flavouring agents together before distilling them. This technique requires special equipment, namely a pot still [14].

During the soaking period (the mixing of the fig spirit with the carob flour), several substances are transferred into the solvent (plant-based alcohol), in addition to the compounds already present in this plant-based alcohol. Those include compounds already present in the plant, like phenolics, and compounds derived from the processing of raw materials, such as the furanics formed during the different thermal treatments of the raw materials (roasting of carob pulp in oven or distillation of dried fruit) due to the thermal degradation of plant sugars [9,15].

Phenolic substances have beneficial biological effects on human health, such as reduction of cardiovascular disease incidence, prevention of some types of cancers, and enhancement of antioxidant status of plasma in humans due to the use of antioxidative therapies that decrease the reactive oxygen species levels associated with neoplasia, atherosclerosis and neurodegenerative diseases, among others [16]. Previous works highlighted the potential of carob pulp as a functional ingredient, partly due to the presence of polyphenols and pinitol, bioactive compounds with AC, and the ability to inhibit certain enzymes such as α-amylase, α-glucosidase and acetylcholinesterase [7,17,18]. On the other hand, furanic compounds such as furfural and 5-hydroxymethyl furfural (HMF), present in carob liqueurs, have toxic effects [9]. The biotransformation of phenolic and furanic compounds elicited by low gastric pH, the presence of digestive enzymes and the microorganisms forming the intestinal flora could change the bioavailability and stability of these compounds, and thus affect greatly the potential bioactivity [3,19,20]. The final chemical composition and biological properties depend on the absorption, metabolism, distribution and excretion of the compounds within the body and the reducing properties of the resulting metabolites [21]. Previous investigations showed that the carob matrix, composed of dietary nutrients such as carbohydrates, dietary soluble fibre, lipid fraction, minerals, proteins and others, in which polyphenols are located, has been an important factor in their stability and digestibility, and consequently their bioaccessibility [4,22].

In this context, the objective of the current study was to determine the influence of the different traditional extraction methods (maceration, infusion, percolation and distillation) used in carob liqueur elaboration on the chemical composition and antioxidant and enzyme inhibitory capacities of the beverages, as well as how in vitro gastrointestinal digestion of the liqueurs affects the stability of the mentioned parameters. For this purpose, the total contents of flavonoids (TFC) and phenolics (TPC), and the AC by using the methods of Trolox equivalent antioxidant capacity (TEAC) and the oxygen radical absorption capacity (ORAC) were analysed in carob liqueurs before (initial) and after the gastrointestinal process (gastric and intestinal phases). Also, the enzyme inhibitory capacity was tested directly in the undigested liqueur and liqueur after the gastric and intestinal phases of the gastrointestinal digestion process, to evaluate the inhibition of acetylcholinesterase (AChE) and tyrosinase, the main enzymes associated with Alzheimer’s [23] and Parkinson´s [24] neurodegenerative diseases, respectively; and of the enzymes linked with type 2 diabetes mellitus [25], α-amylase and α-glucosidase. Furthermore, the qualitative and quantitative phytochemical profile of carob liqueurs was determined using a high-performance liquid chromatography method coupled with photodiode array detection (HPLC-PDA).

## 2. Materials and Methods

### 2.1. Reagents

2,2′-Azino-bis (3-ethylbenzothiazoline-6-sulfonic acid) diammonium salt (ABTS) tablets, potassium persulfate (K_2_S_2_O_8_), 5,5’-dithiobis(2-nitrobenzoic acid) (DTNB), 3,5-dinitrosalicylic acid (DNS) reagent, di-sodium hydrogen phosphate anhydrous (Na_2_HPO_4_), 3,4-dihydroxy-L-phenylalanine (L-DOPA), acetylthiocholine iodide (ATCI), quercetin, acarbose, galantamine, α-amylase type VI-B: from porcine pancreas (EC 3.2.1.1), α-glucosidase type I from *Saccharomyces cerevisiae* (EC 3.2.1.20), acetylcholinesterase (AChE) from *Electrophorus electricus* (electric eel, EC 3.1.1.7, Type VIS), kojic acid, *p*-nitrophenyl-α-D-glucopyranoside (pNPG) and tyrosinase (EC 1.14.18.1) from mushroom were supplied by Sigma-Aldrich (Poole, UK). Gallic acid (GA), sodium chloride (NaCl) and aluminium chloride anhydrous (AlCl_3_) were supplied by Fluka (Steinheim, Germany). (±)-6-Hydroxy-2,5,7,8-tetramethylchromane-2-carboxylic acid (Trolox), 2,2’-azobis(2-methylpropionamidine) dihydrochloride (AAPH) and fluorescein were purchased from Acros Organics (Geel, Belgium). Folin–Ciocalteu reagent (FC reagent), starch from potato soluble and sodium acetate (CH_3_COONa) were acquired from Panreac (Barcelona, Spain). Absolute ethanol was supplied by Fisher Scientific (Manchester, UK). Sodium di-hydrogen phosphate monohydrate (NaH_2_PO_4_·H_2_O) was provided by Merck (Darmstadt, Germany). Sodium carbonate (Na_2_CO_3_) was purchased from José Manuel Gomes dos Santos, Lda (Odivelas, Portugal).

### 2.2. Production and Digestion Processes of Carob Liqueurs

For the experiments, we used a commercial roasted carob pulp flour from plurivarietal *Ceratonia siliqua* L. fruits (Industrial Farense Lda, Faro, Portugal) with the following nutritional information per 100 g of flour: energetic value 340 Kcal, lipids 0.8 g (saturated 0.2 g), carbohydrates 37 g (sugars 26 g), proteins 4 g, sodium 0.1 g and fibre 9 g.

**Figure 1:**
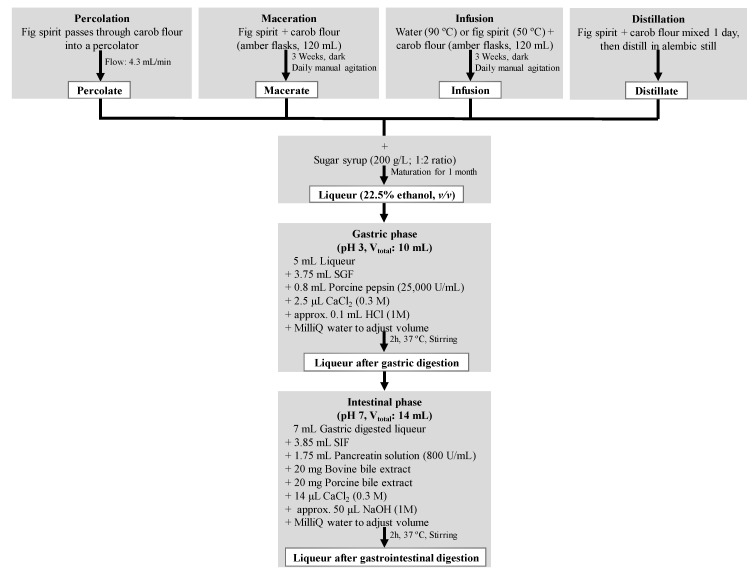
Schematic representation of the liqueur production, by different extraction methods, and the gastric and intestinal phases of the in vitro digestion process, with the respective constituents (such as simulated gastric (SGF) and intestinal (SIF) fluids) and conditions used.

To produce the liqueur, carob pulp was mixed with fig spirit (45% *v*/*v*, Santa Catarina Cooperative, Fonte do Bispo, Portugal) or soft water (Fastio, Gerês, Portugal) at a proportion of 5% *w*/*v*, according to the various extraction methods studied. The steps of liqueur production are depicted in Figure 1.

**Figure 2:**
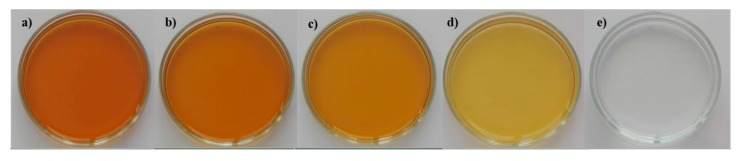
Carob liqueurs obtained by different extraction methods: hydro-alcoholic infusion (**a**), maceration (**b**), percolation (**c**), aqueous infusion (**d**) and distillation (**e**).

Figure 2 shows the liqueurs obtained with different extraction methods, illustrating the colour differences observed.

The obtained liqueurs (a total of 15 samples: five extractive methods x three replicates) were subjected to a simulated gastrointestinal digestion process, as detailed in Figure 1. The in vitro digestion consisted of an initial gastric phase followed by an intestinal phase using different enzymes (pepsin and pancreatin, respectively), pH (3 and 7, respectively) and simulated fluids (gastric (SGF) and intestinal (SIF), respectively) prepared with different proportions of mineral salts (KCl, KH_2_PO_4_, NaHCO_3_, NaCl, MgCl_2_·6H_2_O and (NH_4_)_2_CO_3_), as previously described by Minekus et al. [26], with the modifications of Jara-Palacios et al. [27] (Figure 1). Immediately after each phase, aliquots of gastric and gastrointestinal digested liqueurs were taken, stored at −20 °C and analysed within two weeks. The digestion procedure was repeated three times for each of the 15 liqueur samples.

### 2.3. Identification and Quantification of the Phenolic and Furanic Compounds by HPLC-PDA Analysis

Liqueurs phenolic and furanic compositions were analysed following the HPLC procedure of Rodríguez Solana et al. [9] with slight modifications. Briefly, 20 μL of the sample or standard were analysed using a HPLC-PDA system (Varian 920-LC) and a Kromasil 100 Å pore size C18 column (250 mm length × 4.6 mm i.d. and 10 μm of particle size). The solvent mixture system contained 0.1% formic acid in water (A) and acetonitrile (B) with a flow rate of 1 mL/min. Separation was achieved using a gradient flow as follows: 0 min: 92% A and 8% B; 5 min: 90% A and 10% B; 10 min: 80% A and 20% B; 20 min: 70% A and 30% B; 30 min: 10% A and 90% B, and 35 min: 92% A and 8% B. Detection was carried out by using a photodiode array detector at 280 nm. Qualitative identification of gallic acid, furfural and 5-hydroxymethylfurfural in samples was based on the comparison of UV spectra and retention time in chromatograms with authentic standards (Figures S1–S6). Quantification was determined using external calibration preparing calibration curves of standard solutions in methanol (Table 1). The concentrations were calculated by taking into account both the dilutions inherent to the digestion process, and those necessary to have the results within the range of the linear calibration curve. All samples were filtered through a 0.2-μm pore cellulose acetate membrane (VWR International, Westchester, PA, USA) before the analysis, and the determinations were performed in triplicate. The different parameters of the calibration curves, coefficient of determinations (*r*^2^) and limits of detection (LOD) and quantification (LOQ) are shown in Table 1. Both limits are calculated based on three (LOD) or 10 (LOQ) times the standard deviation of the calibration curve and divided by the slope.

### 2.4. Total Phenolic (TPC) and Flavonoid (TFC) Contents, and Antioxidant Capacities by Trolox Equivalent Antioxidant Capacity (TEAC) and Oxygen Radical Absorption Capacity (ORAC) Assays

#### 2.4.1. TPC by Folin-Ciocalteu Method

The TPC of different carob liqueurs before and after the gastric and intestinal phases was measured using the Folin-Ciocalteu (FC) colorimetric method [28]. Briefly, 250 μL of FC reagent were mixed with 50 μL of properly diluted carob liqueur, 20% ethanol/water solution (blank) or GA (standard) at different concentrations (50–300 mg/L). Then, 750 μL of Na_2_CO_3_ (7%, *w*/*v*) were added and the mixture was topped up to 5 mL with pure water. The reaction was incubated at room temperature in the dark for 2 h. The absorbance was measured at 760 nm using a 1-cm quartz cuvette and a T70 + UV–Visible Spectrophotometer (PG Instruments Ltd., Leicester, UK). The quantification of the samples was carried out using a calibration curve with known concentrations of GA and the results were expressed as milligrams of GA equivalents (GAE) per litre of liqueur (mg_GAE_/L).

#### 2.4.2. TFC by Aluminium Chloride Method

TFC of each sample was estimated using an adapted aluminium chloride (AlCl_3_) colorimetric method [29]. In short, 500 μL of properly diluted carob liqueur, 20% ethanol/water solution (blank) or quercetin (standard) at different concentrations (0.02–0.25 mM) were mixed with 100 µL of 1% (*w*/*v*) AlCl_3_ solution, 100 µL of 1 M sodium acetate solution and 1.5 mL of 80% ethanol/water. The absorbance was read at 415 nm after 30 min of incubation using a T70 + UV-Visible Spectrophotometer (PG Instruments Ltd.). The quantification of the samples was carried out using a calibration curve with known concentrations of quercetin and the results were expressed as milligrams of quercetin equivalents (QE) per litre of liqueur (mg_QE_/L).

#### 2.4.3. TEAC Method (ABTS Free Radical Scavenging Assay)

The TEAC assay was performed according to the method proposed by Re et al. [30]. The ABTS^•+^ was generated by reacting an ABTS tablet with a 2.45 mM potassium persulfate solution to give a final concentration of 7 mM. The mixture was stored in the dark at room temperature for 12–16 h before use. The ABTS^•+^ working solution was prepared by diluting the previous solution with water until reaching an initial absorption value of 0.70 ± 0.02 at 734 nm. Briefly, 10 μL of properly diluted sample, 20% ethanol/water solution (blank) or Trolox (standard) at different concentrations (0.1–0.5 mM) were placed in a 96-well microplate. The reaction began after the addition of 190 μL ABTS^•+^ working solution. The absorbance was read at 734 nm using a Tecan Infinite M200 microplate reader (Tecan, Männedorf, Switzerland). The results were expressed as millimoles of Trolox equivalents (TE) per litre of liqueur (mmol_TE_/L).

#### 2.4.4. ORAC Assay

The ORAC assay was carried out according to the protocol proposed by Gillespie et al. [31], in which fluorescein is used as the fluorescent probe and AAPH as peroxyl radical generator. The ORAC assay was carried out in black round-bottomed 96-well microplates. A volume of 150 μL of 0.08 mM fluorescein was mixed with 25 μL of properly diluted sample, 20% ethanol/water solution (blank) or Trolox (standard) at different concentrations (6.25–50 µM). The microplate was incubated at 37 °C for 10 min. After the addition of 25 μL of AAPH (150 μM) to each well, the kinetic read started at once. Fluorescence was measured every 5 min for 90 min with an excitation wavelength at 485 nm and an emission wavelength at 530 nm in a Tecan Infinite M200 microplate reader. The ORAC values were calculated using the quadratic regression equation obtained from concentrations of Trolox stock solutions and the area under the curve (AUC). The results were expressed as millimoles of Trolox equivalents (TE) per litre of liqueur (mmol_TE_/L).

### 2.5. Enzyme Inhibitory Capacities

#### 2.5.1. Acetylcholinesterase (AChE) Activity Method

The AChE inhibitory activity of carob liqueurs was determined using the Ellman’s method [32] with some modifications [33]. Briefly, 125 μL DTNB 3 mM was mixed with 25 μL ATCI 15 mM, the AChE substrate, 50 μL of sodium phosphate buffer (100 mM, pH 8), and 25 μL of each carob liqueur, buffer (blank) or galantamine (positive control, 25 µg/mL), in a 96-well microplate. Finally, 25 μL of AChE (0.28 U/mL) were added and the absorbance was measured immediately, and after 5 min at 405 nm. The hydrolysis of ATCI was monitored by the formation of the yellow 5-thio-2-nitrobenzoate (TNB) anion as a result of the reaction of DTNB with thiocholines, catalysed by enzymes. A control without liqueur sample was performed and the AChE inhibitory activity was expressed as a percentage of inhibition.

#### 2.5.2. Tyrosinase Inhibition

Tyrosinase activity was measured following Masuda et al. [34] with slight modifications. Briefly, 80 μL of sodium phosphate buffer (20 mM, pH 6.8) were mixed with 40 μL of tyrosinase solution (46 U/mL prepared in buffer) and 40 μL of carob liqueur, sodium phosphate buffer (blank) or kojic acid (positive control, 200 mg/L). The blank_control_ and the blank_sample_ (used to eliminate some interference of the liqueur colour)_,_ without the enzyme, was prepared by adding 160 μL of buffer or 160 μL of buffer and 40 μL of the sample. The mixture was incubated at room temperature for 10 min. Then, 40 μL of L-3,4-dihydroxyphenylalanine (L-DOPA, substrate) were added and the mixture was incubated at room temperature for 10 min. After that period, the absorbance was measured at 475 nm. A control without liqueur sample was tested and the tyrosinase inhibitory activity was expressed as a percentage of inhibition.

#### 2.5.3. α-Amylase Assay

The assay for measuring amylase activity was performed according to the method described by Ali et al. [35]. A volume of 40 μL of sample, sodium phosphate buffer (20 mM, pH 6.9 with 6 mM NaCl) (blank) or acarbose (positive control: 1 mg/mL), 160 μL of sodium phosphate buffer and 200 μL of α-amylase enzyme solution (4 U/mL in buffer) was mixed in test tubes and incubated at room temperature for 5 min. Then, 400 μL of potato starch solution (0.5% *w*/*v* in buffer) were added and incubated for 3 min at room temperature. Finally, 400 μL of DNS reagent were added, and the mixture was incubated for 15 min in a water bath at 85 °C. After cooling at room temperature, 50 μL of the mixture were transferred to 96-well microplates and diluted with 150 μL of distilled water. The absorbance was read at 540 nm and the results were expressed as a percentage of inhibition.

#### 2.5.4. α-Glucosidase Assay

All samples were assayed by the α-glucosidase inhibitory method, adapted from Kwon et al. [36], wherein the *p*NPG is hydrolysed by α-glucosidase to release *p*-nitrophenol, which is detected at 405 nm. For that, 50 μL of sample, sodium phosphate buffer (control experiment or blank; 100 mM, pH 6.9) or acarbose (positive control; 1 mg/mL) were mixed with 100 μL of α-glucosidase solution (1.0 U/mL) in a 96-well microplate. The mixture was incubated at room temperature for 10 min and, after incubation, the absorbance was measured at 405 nm. Then, 50 μL of the substrate *p*NPG (5 mM in buffer) were added to each well at room temperature and the absorbance of the mixture was measured immediately. The α-glucosidase inhibitory activity was determined by measuring the effect on the enzyme reaction rate after adding liqueur or acarbose, compared with the control without the sample.

### 2.6. Statistical Analysis

Statistical analysis was carried out using Statistix v9 (Analytical Software, Tallahassee, FL, USA). Data were evaluated by one-way (enzyme inhibitory capacities of liqueurs in each digestion phase) and two-way (remaining parameters in samples) analysis of variance (ANOVA). When ANOVA showed significant differences (*p* < 0.05) separation of means was achieved using Fisher’s least significant difference at *p* = 0.05. Pearson’s correlations (*r)* were performed to assess the relationships among the different parameters studied, and correlations with *p* < 0.05 were considered statistically significant.

The compositions of undigested and digested (gastric and intestinal phases) liqueurs prepared by maceration, infusion, percolation and distillation methods were compared using principal component analysis (PCA) (XLSTAT Software, Addinsoft, New York, NY, USA).

## 3. Results and Discussion

### 3.1. Stability of the Phenolic and Furanic Compounds of Carob Liqueurs during Simulated In Vitro Gastrointestinal Digestion

A previous study showed that gallic acid (GA), furfural and HMF are the main chemical compounds found in liquors prepared with roasted carob flour [9]. In the present study, the concentrations of these compounds in carob liqueurs elaborated by five extraction methods (percolation, maceration, aqueous or hydro-alcoholic infusions and distillation), before and after the digestion process, are shown in Table 2. Gallic acid, the main phenolic compound identified in previous works with carob products [9,37,38], was extracted in higher quantities in hydro-alcoholic infusion and maceration methods, followed by percolation and aqueous infusion. The short contact time between solvent and solute used in percolation procedure or the use of a single solvent cycle going through the carob flour was insufficient to extract greater amounts of this compound. In the case of aqueous infusion, water (even at elevated temperatures) was a poor solvent to extract this compound. This is in accordance with the results obtained by Daneshfar et al. [39] and Kallithraka et al. [40], which studied the solubility of GA in different solvents and found that 100% or 75% ethanol was better than water. As for the distillation technique, ethanol and water are carriers of a huge number of volatile compounds, so it is normal for the extraction of GA not to occur since it is not a volatile compound.

The conditions of the gastric hydrolytic process (low pH and enzyme action) may favour the release of GA bound to other structures present in the food matrix such as carbohydrates (e.g., gallotannins: monogalloyl-glucoside, digalloyl-glucoside, tetragalloyl-glucoside and tetragalloyl-glucoside), and thus increasing the content of this compound [41]. This is consistent with the slight increase in GA content found in liqueurs obtained by aqueous infusion (recovery of 106.28%). A similar trend was observed in samples of red wine subjected to gastric conditions, where the concentration of GA significantly increased in relation to the initial sample (*p* < 0.01) [41]. However, the concentration of this compound in liqueurs, elaborated by hydro-alcoholic infusion and maceration, showed no significant differences (*p* < 0.05) before and after the gastric digestion, with recoveries of 98.24% and 97.30%, respectively. Therefore, the GA bioavailable in these liqueurs is probably already in its free form, since the initial roasting process carried out on carob pulp favours the release of GA bonded to complex structures [9]. In accordance with these results, Ydjedd et al. [4] found that phenolics released from nonencapsulated carob pulp extracts showed high stability after the gastric step. On the contrary, the recovery decreased in liqueurs obtained by percolation, and in other works of a carob pulp product from Cyprus [5], and in Spanish carob flour extract [22], with recoveries of 90.53%, 83% and 68.28%, respectively.

The modifications in pH after the gastrointestinal digestion, stomach (pH = 3) and intestinal (pH = 7) phases, may cause appreciable alterations in the structure and physicochemical properties of the bioactive compounds, and/or different interactions resulting in oxidation, precipitation of phenolics (e.g., tannins) with enzymes present in the digestive mixture, and interactions with other components such as polysaccharides, etc. [4]. Gallic acid was almost entirely degraded in liqueurs made by percolation and aqueous infusion. Similarly, in nonencapsulated ripe carob pulp extracts (with recoveries from 149.6% to 0.6%, for gastric and intestinal phases, respectively) [4], and extracts from different fruit seeds [42], the GA was completely digested after the digestion process. However, no significant differences were found after the gastrointestinal digestion in hydro-alcoholic infusion liqueurs (recovery of 91.19%), and a slightly significant decrease was observed in maceration liqueurs (recovery of 87.47%). An intermediate behaviour was observed in previous works on carob extracts, with recoveries of 50% [5] and 47.70% [22]. These different performances can be associated with the food matrix present in carob liqueurs obtained from the diverse extraction processes [5,22,41]. Highest degradation of GA was observed in liqueurs obtained by percolation and aqueous infusion. This can be explained by the fact that both methods provided less complex liqueur matrices and, therefore, gallic acid may be more susceptible to degradation. However, the higher content of sugars and soluble fibre present in the matrix of liqueurs elaborated by hydro-alcoholic infusion and maceration could act as a protective barrier to GA degradation [22].

The furanic compounds identified in samples, HMF and furfural, considered toxic to humans, are formed during thermal processing of carob pulp or fig, to produce respectively, the roasted carob pulp (formation of furfural and HMF) and the fig spirit (furfural) used in liqueur elaboration [9]. In carob liqueurs, HMF showed similar extraction behaviour to that of GA (high Pearson correlation value, Table 3). The highest HMF contents were found in liqueurs obtained by maceration and hydro-alcoholic infusion, followed by percolation and finally aqueous infusion. As can be deduced from the results, the extraction of this compound is favoured by the direct contact between solvent and solute during the soaking of both raw materials in percolation, maceration and infusion methods. Whereas during distillation, where the volatilization of compounds takes place, only trace amounts were extracted. The HMF concentrations after the gastric phase of the digestion process did not differ significantly (*p* < 0.05) from those of the initial liqueurs. However, after the intestinal phase, the HMF bioavailability reduced to recoveries of 59.52%, 72.18%, 84.73 and 89.64% for liqueurs elaborated by percolation, maceration, hydro-alcoholic infusion, and aqueous infusion, respectively. According to Hamzalıoğlu and Gökmen [43], under intestinal conditions this reduction is explained by the high reactivity of HMF towards amino and sulfhydryl groups present in amino acids derived from protein hydrolysis during gastric digestion, and with the subsequent formation of Michael adducts and Schiff bases. The concentration of furfural found in liqueurs at the end of the digestion process showed no significant differences among the infusions, maceration and percolation methods. This is not surprising, since this compound arises from the fig spirit used in the liqueur preparation (Table 2), which is used at the same volume in the different extraction processes. The highest concentrations found in samples obtained by distillation, may be related to the degradation of sugars produced during the distillation of the fig spirit with the carob pulp flour to extract the carob volatiles [10].

The concentrations of both furanic compounds found after the digestion process are on the order of those considered safe, as shown previously [9]. Therefore, the moderate intake of these liqueurs does not represent any danger to human health.

### 3.2. Evaluation of the Total Phenolic and Flavonoid Contents and Antioxidant Capacity of Carob Liqueurs

**Figure 3:**
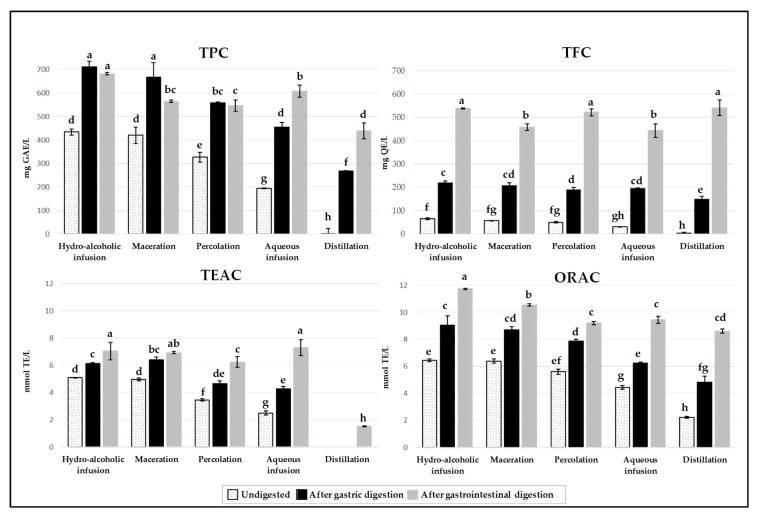
Total phenolic (TPC) and flavonoid (TFC) contents and antioxidant capacity measured by Trolox equivalent antioxidant capacity (TEAC) and oxygen radical absorbance capacity (ORAC) assays of carob liqueurs obtained with different extraction methods and subjected to gastrointestinal digestion. In each graph (TPC, TFC, TEAC or ORAC), values marked with different letters indicate a significant difference (*p* < 0.05).

Phenolics, particularly flavonoids, are compounds linked to the antioxidant capacity (AC) of plant extracts and already found in different carob products [4,5,6]. As can be seen in Table 3, high correlations were found between the total phenolic content (TPC) and the AC by two methods based on different mechanisms used to evaluate the antioxidant effect of phenolic compounds, the ORAC and TEAC assays. The values found for each parameter are significantly different among undigested and/or digested carob liqueurs, based on the type of extraction method used to produce the liqueur (Figure 3). Even though the liquid matrix of these samples already possesses compounds (such as natural antioxidants) promptly bio-accessible and ready to exert their beneficial effects on the gastrointestinal tract, the behaviour after each digestion phase will depend on the matrix composition, which varies according to the extraction conditions (technique) used during liqueur elaboration [41]. After the gastric and intestinal phases, the values of the mentioned parameters are significantly higher in comparison to the initial liqueur, regardless the extraction method used in the liqueur elaboration (Figure 3). Similar results were observed in commercial fruit juices [19,20]. In these works, the authors attributed the higher AC of digested samples to the biotransformation, by hydrolyzation, of polyphenolic compounds, such as quercetin glycosides to quercetin, in mild alkaline conditions and using digestive enzymes (pepsin and pancreatin). Also, the different processing steps of juice production contributed to such biotransformation, and therefore, to the increase of AC values. Attri et al. (2017) [19] explained that the highest increase in AC after gastrointestinal digestion in pineapple juice, in relation to other juices, can be due to the high sodium concentration present in that sample affecting the release of antioxidants from the food matrix. As can be inferred by the composition of the carob pulp used in this work (0.1 g of sodium), the water used for dilution (4.1 mg/L of sodium), and the fig spirit (another sodium source), as well as the mineral composition found in carob liqueurs in a previous work [44], the presence of sodium can also explain the results obtained after gastrointestinal digestion. A previous study showed that TPC and AC, measured by DPPH and FRAP assays, of different carob pulp products also increased after gastric step, but, decreased after intestinal phase [5]. According to these authors, the increase of TPC during gastric conditions (acidic medium) could be caused by the release of phenolic compounds from the matrix or the increased reactivity of phenolic compounds towards Folin-Ciocalteu reagent.

Regarding the content of flavonoids (TFC) shown in Figure 3, hydro-alcoholic infusion (65 ± 4 mg_QE_/L), maceration (57 ± 0.4 mg_QE_/L) and percolation (50 ± 2 mg_QE_/L) presented significantly higher values, followed by aqueous infusion (31 ± 1 mg_QE_/L) and finally distillation (4 ± 1 mg_QE_/L). For all of them, the content increased significantly after the gastric and intestinal digestions, presenting in the latter case the highest value. Ortega et al. [22] explained that this increase, after the intestinal step of an extract of carob flour, can be due to the release of flavonoids bounded to sugar residues. The mixture of sugar and soluble fibre in the medium could be responsible for the protective effect in the recovery and enhancement of the flavonoids bioaccessibility. The extraction of flavonoids from the complex structures after the digestion, could also explain the high ORAC values found in samples in the present work, since a high correlation between both parameters was observed (Table 3). ORAC values can also be correlated to the number of hydroxyl groups present in A and B rings of flavonoids [45,46]. Although flavonoids are a class of phenolic compounds, a slight correlation was found between TFC and TPC (Table 3). This can be explained, on the one hand, by using an aluminium chloride method that evaluates the TFC, and thus underestimating the content of flavonoids, since it relates to only flavones and flavonols, without including flavanones [47]. On the other hand, the Folin-Ciocalteu reagent can react with diverse reducing nonphenolic substances present in carob pulp, such as amino acids, proteins, sugars, vitamin C and other organic acids, which may lead to the overestimation of the TPC values [4,48,49].

The statistically significant (*p* < 0.05), high correlations found between HMF contents, TEAC (0.741) and TPC (0.598) values, and between TFC, ORAC (0.838) and TPC (0.615), suggest that the contents in flavonoids and HMF contribute most to the variation of AC and TPC in samples. The influence of HMF in TPC and AC by TEAC assay was also previously observed in lime honey [50].

### 3.3. Enzyme Inhibitory Capacities of Carob Liqueurs

**Figure 4:**
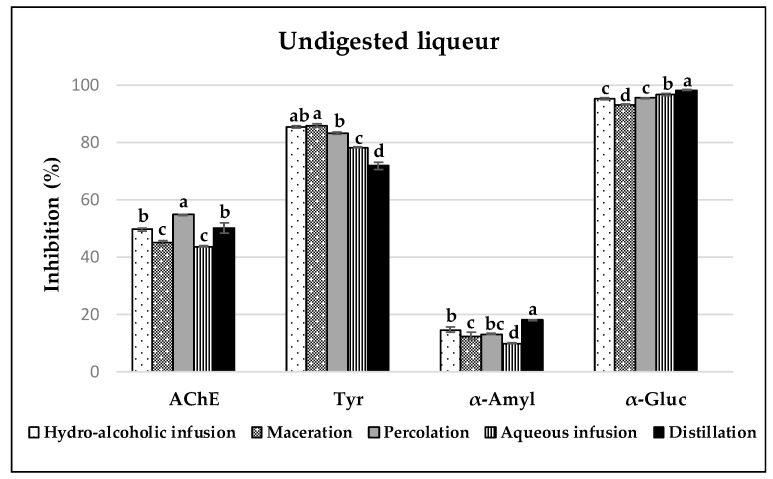
Enzyme [acetylcholinesterase (AchE), tyrosinase (tyr), α-amylase (α-amyl) and α-glucosidase (α-gluc)] inhibitory capacities of undigested carob liqueurs obtained with different extraction methods. For each enzyme (AchE, tyr, α-amyl, or α-gluc) inhibition method, values marked with different letters indicate a significant difference (*p* < 0.05).

**Figure 5:**
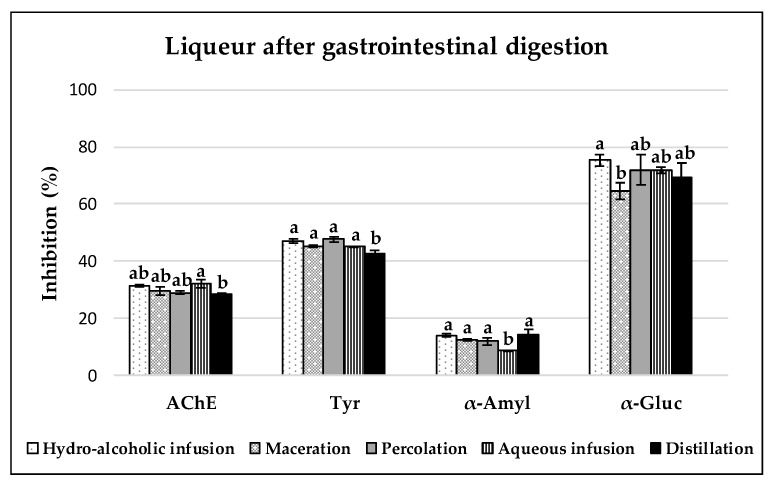
Enzyme [acetylcholinesterase (AchE), tyrosinase (tyr), α-amylase (α-amyl) and α-glucosidase (α-gluc)] inhibitory capacities of gastrointestinal digested carob liqueurs obtained with different extraction methods. For each enzyme (AchE, tyr, α-amyl, or α-gluc) inhibition method, values marked with different letters indicate a significant difference (*p* < 0.05).

All carob liqueurs studied presented inhibitory capacities against the enzymes AchE, tyrosinase, α-amylase and α-glucosidase; however, different patterns were observed before (Figure 4) and after the digestion (Figure 5) of samples, depending on the enzyme studied. The higher inhibition percentages were obtained for α-glucosidase and tyrosinase, followed by AchE, and finally α-amylase.

#### 3.3.1. Acetylcholinesterase (AchE) Inhibition

In the healthy brain, AchE is the predominant cholinesterase (80%). This enzyme is responsible for the hydrolysis of the neurotransmitter acetylcholine in choline and an acetyl group, and is mainly distributed throughout the synapses of the brain and neuromuscular junctions [50,51]. The accumulation of malignant β-amyloid plaques in the brain tissues of patients with Alzheimer’s disease (AD) has been proven to be in association with increased amounts of AchE [51]. Therefore, acetylcholinesterase inhibitors (AchEIs) are expected to be useful agents for augmenting cholinergic activity, and thus preventing the development of neurodegenerative diseases such as AD [52]. Because synthetic cholinesterase inhibitors are limited in use due to their adverse side effects, such as gastrointestinal disturbances and bioavailability problems, there is still a need for the development of more potent agents, either natural products or synthetic analogues, with minimal side effects for the treatment of AD [53,54]. In this sense, the carob liqueurs, before and after the gastrointestinal digestion, were analysed and showed moderate activity of this enzyme, with values below that of the positive control, galantamine (25 µg/mL, 72.96 ± 0.26%). Similar results to those of liqueurs prepared by aqueous infusion (43.58 ± 0.14%) and maceration (44.90 ± 0.95%) were observed in dilutions (10 mg/mL; 37% of inhibition) prepared from carob pulp decoctions [7]. Samples prepared by percolation (54.74 ± 0.12%) presented the highest AchE inhibition, followed by liqueurs obtained by distillation (50.02 ± 1.83%) and hydro-alcoholic infusion (49.62 ± 0.59%). The inhibitory capacity of this enzyme was negatively correlated with TPC, TFC and with the AC by ORAC, while no correlation was found with the compounds identified by HPLC-PDA (Table 3). Similar inhibition was found in *Tanacetum haussknechtii* essential oil (51.20%), and the inhibition was related to the presence of monoterpenoids with pinene and camphene skeletons [55]. The methanol and ethyl acetate extracts of this plant (19.20–37.10%) presented lower inhibition, which was associated with caffeoylquinic acid and its derivatives as well as flavonoid constituents. However, dilutions of 10 mg/mL from decoctions of carob leaves (88 ± 6%) and carob stem bark (87 ± 4%) [7], as well as dilutions (1 mg/mL; 75.60 ± 0.41%) of methanol extracts from carob leaves [56], showed higher inhibitions. It should be noted that, despite the significantly different inhibition percentages found in the different initial liqueurs, no significant differences were found after the gastrointestinal process (*p* < 0.05).

#### 3.3.2. Tyrosinase Inhibition

Tyrosinase is a polyphenol oxidase with a dinuclear copper active site involved in some dermatological disorders associated with the excessive formation of mammalian melanin pigments (melanin hyperpigmentation), the enzymatic browning of damaged fruits and vegetables during postharvest handling and processing, and the neurodegeneration associated with Parkinson’s disease [57,58]. These undesirable phenomena can be partly prevented with tyrosinase inhibitors, chemical agents capable of reducing the activity of this enzyme. Kojic acid is a chemical used in whitening cosmetics, and is usually used as a positive control in this enzymatic inhibitory method (200 µg/mL, 93.49 ± 0.29%). However, some adverse effects have been found for this compound, such as severe skin inflammations and chronic, cytotoxic and mutagenic effects. Therefore, the search for new antityrosinase agents of natural origin that can replace its action, such as plant-based secondary metabolites, is of the utmost interest [51]. Within this category, polyphenols are the largest group, with GA being the one with the highest inhibitory activity [59]. Undigested liqueurs elaborated with the different extraction methods presented potent tyrosinase activity. Hydro-alcoholic infusion (85.28 ± 0.32%), maceration (85.83 ± 0.49%) and percolation (83.19 ± 0.36%) liqueurs showed the higher inhibitions. As mentioned above, this may be associated with the concentrations of GA found before and after gastric digestion (Table 2) and corroborated by the correlations found between this compound and the antityrosinase activity (Table 3). Lower concentrations of methanol extracts from carob leaves showed higher inhibitions (200 µg/mL, approx. 90%; 400 µg/mL, approx. 125%) [60]. Finally, the activity became moderate for all liqueurs after the gastrointestinal digestion, and, in general, it can be related to the degradation of GA in samples.

#### 3.3.3. α-Amylase and α-Glucosidase Inhibition

Diabetes mellitus is currently one of the most costly and burdensome chronic diseases and it is also linked to several other diseases. Its incidence in the world is increasing, affecting close to 5% of the global population. About 90% of diabetics have non-insulin-dependent diabetes mellitus (type 2 diabetes) [61]. This type of diabetes is characterized by chronic hyperglycaemia and gross derangement in carbohydrate, lipid and protein metabolism, due to a deficiency in insulin secretion and/or action [51,61,62]. Inhibiting or delaying the digestion and absorption of carbohydrates from the intestinal tract, and thus modulating the elevation of postprandial glucose in the blood, is a more important factor that can effectively reduce the symptoms. For this purpose, the inhibition of α-glucosidase (located in the mucosal brush border of the small intestine) and α-amylase (a salivary or pancreatic enzyme) is one of the most effective therapeutic methods [51]. α-Amylase plays an important role in the early breakdown of complex carbohydrates and the hydrolysis of large and insoluble starch molecules to simple absorbable molecules, ultimately glucose and maltose; α-glucosidase is a key enzyme to metabolize nonabsorbable oligosaccharides into absorbable monosaccharides in the small intestine, the end step of digestion of starch and disaccharides abundant in the human diet [62]. Certain drugs have strong inhibitory capacities against both enzymes, but present side effects such as abdominal distention, flatulence, meteorism and possibly diarrhoea. These side effects could be caused by excessive inhibition of pancreatic α-amylase, resulting in the abnormal bacterial fermentation of undigested carbohydrates in the colon [61]. Therefore, the search for new sources, mainly of natural origin, that reduce the activity of both enzymes is of vital importance.

In this work, acarbose (1 mg/mL), a commercially available enzyme inhibitor for type 2 diabetes, was used as a reference, showing an inhibition of 78.34% and 78.33% for α-glucosidase and α-amylase, respectively. All samples exhibited potent (>50%) ability to inhibit the activity of α-glucosidase. After digestion, higher inhibition percentages were found in liqueurs elaborated with the hydro-alcoholic (75.28 ± 2.06%) and aqueous (71.81 ± 1.00%) infusion, and percolation (71.92 ± 5.25%) methods. On the other hand, the liqueurs before and after the digestive process showed low activity (<30%) against α-amylase. Dilutions of 10 mg/mL from decoctions of carob pulp presented lower values for α-glucosidase (22 ± 2%) and α-amylase (7 ± 0%) inhibitions; however, a similar proportion of enzyme inhibitions was shown [7]. According to a previous work [63], the strong ability to inhibit the activity of α-glucosidase, together with a low inhibitory effect against α-amylase, are ideal conditions for the management of postprandial hyperglycaemia with minimal side effects. In addition, the combination of the AC (which leads to the alleviation of oxidative stress produced by the chronic hyperglycaemia in diabetes, thus preventing or reversing diabetic complications), and the mentioned proportion of inhibitory capacities against both enzymes make samples a more effective antidiabetic agent. In this work, these antioxidant and enzyme inhibitor capacities were present in the carob liqueurs after gastrointestinal digestion.

No strong correlations were found between enzyme inhibitory capacities and the compounds identified in this work (Table 3). According to Christou et al. [17], D-pinitol is a compound present in the fruit of *C. siliqua* and it is known to be related to the antidiabetic activity in samples.

### 3.4. Principal Component Analysis

The biplot of the two main principal components (PC1 and PC2) characterized by the enzyme (AChE, α-glucosidase, α-amylase and tyrosinase) inhibitory capacities, the TPC, TFC, GA, HMF and furfural contents, and the ORAC and TEAC antioxidant capacities of carob liqueurs obtained by aqueous and hydro-alcoholic infusions, distillation, maceration and percolation methods, and subjected to gastrointestinal digestion, can be observed in Figure 5. The first principal component (PC1) was mostly characterized positively by the variables AChE (0.708) and tyrosinase (0.483) inhibitions, TFC (0.817), TPC (0.711), ORAC (0.888), TEAC (0.514) and furfural (0.489), and accounting for 45.60% of the total variance; while GA (0.813) and HMF (0.946) dominate in the second principal component (PC2), representing 29.17% of the total variance.

**Figure 6:**
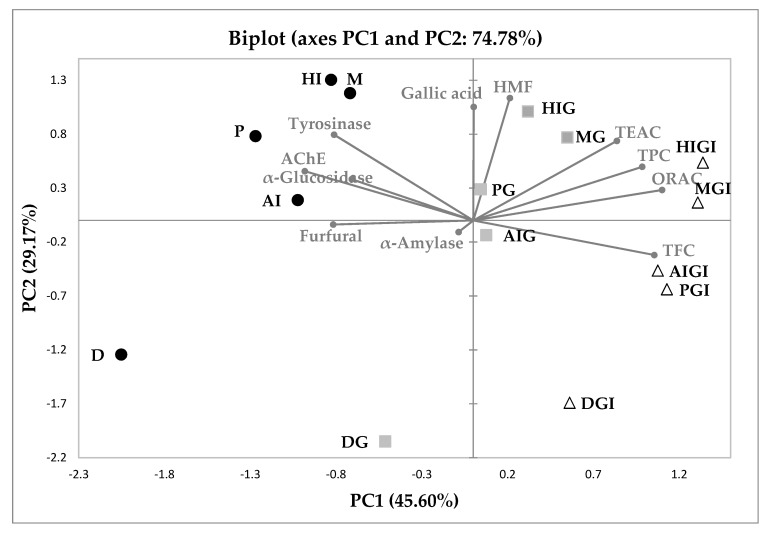
Principal component (PC) analysis plot of gallic acid, furfural, 5-hydroxymethyl furfural (HMF), antioxidant capacity [Trolox Equivalent Antioxidant Capacity (TEAC) and Oxygen Radical Absorption Capacity (ORAC) assays], total phenolic (TPC) and flavonoid (TFC) contents and inhibition of enzymatic (α-amylase, α-glucosidase, acetylcholinesterase (AChE) and tyrosinase) capacities of carob liqueurs (circles) obtained by aqueous (AI) and hydro-alcoholic (HI) infusions, distillation (D), maceration (M) and percolation (P), and subjected to gastric (squares) and gastrointestinal (triangles) digestions.

Overall, a separation of samples into three groups was found according to the digestion treatment (Figure 6). The first group, on the right side of PC1 (the first and fourth quadrant), is composed of samples after gastrointestinal digestion. These samples showed the highest values for TPC, AC (ORAC and TEAC assays), TFC, and HMF concentrations, variables clustered together on the positive side of PC1. The second group, in the central part of the graph (mainly the positive part of PC1), is constituted by liqueurs after gastric phase. These samples presented values of AC (TEAC and ORAC) and TPC close to those of gastrointestinal digestion samples, as well as concentrations of GA and HMF close to the undigested samples. The third group, in the negative side of PC1 and mainly in the positive part of PC2, is formed by undigested liqueurs. The inhibitions of the enzymes, α-glucosidase, AChE and tyrosinase, and the concentrations of GA and HMF, are the parameters that better define these samples, due to both the highest proportions found and the higher initial concentration of liqueur (50 mg/mL).

The main findings obtained in hydro-alcoholic infusion and maceration liqueurs were very similar, and therefore, both samples are positioned close to each other in the graph. The same applies in the case of liqueurs made by aqueous infusion and percolation methods.

On the other hand, it is important to point out the different behaviour observed in the liqueur obtained by distillation, both before and after gastrointestinal digestion. The results differ from the rest, hence, in the graph they are located at the bottom of the chart, on the negative side of PC2, away from the other liqueurs. These results are justified by the fact that distillation is mainly used for the concentration of volatiles, so that the aroma in the sample is extoled. The other methods are used, not only for the extraction of volatiles, but also to obtain other components (such as phenolics), to give full-bodied liqueurs and deep structure. Therefore, these different food matrices would be the reason for the differences in results.

## 4. Conclusions

In this study, the traditional extraction techniques used in liqueur elaboration influenced the chemical composition and antioxidant and enzyme inhibition capacities of carob liqueurs before and after the different phases (gastric and intestinal) of the digestion process. The results obtained indicate that hydro-alcoholic infusion, closely followed by maceration, allowed the strongest AC, TPC and TFC, as well as the highest bioavailability of GA, even after the digestive process. On the contrary, in general, distillation was the method with the lowest values since it is a specific technique for volatile extraction. The results also showed that the furanic content present in liqueurs does not involve any health risk. Despite the initial differences in enzyme inhibitory capacities found in samples, after the gastrointestinal digestion, similar inhibitory capacities were found for all carob liqueurs regardless of the extraction method used in their elaboration. Digested liqueurs showed potent and low inhibition to α-glucosidase and α-amylase, respectively, enzymes related to diabetes type 2; and moderate inhibition to tyrosinase and acetylcholinesterase, enzymes linked with neurodegenerative diseases. The results found in this work suggest that a light-to-moderate carob liqueur consumption might contribute to prevent the oxidative stress, as well as provide beneficial effects on the risk of type 2 diabetes mellitus and neurodegenerative diseases. Nevertheless, this is a preliminary in vitro digestion study and further in vivo analysis must be carried out to determine the bioavailability of compounds present in carob liqueurs with the corresponding protective effect on human health.

## Figures and Tables

**Table 1:** Compound name, linear parameters and analytical limits of the high-performance liquid chromatography method coupled with photodiode array detection (HPLC-PDA) method for the quantification of the main compounds identified in carob liqueurs.

Compound	RT (min)	Calibration Range (mg/L)	Regression Equation *y* = a*x* + b	*r* ^2^	LOD (mg/L)	LOQ (mg/L)
Gallic acid	4.75	5–55	*y* = 33.901*x* − 15.493	0.999	1.97	6.58
HMF	6.45	1–35	*y* = 123.37*x* + 63.257	0.999	1.26	4.20
Furfural	9.65	1–20	*y* = 149.39*x* + 19.349	1	0.40	1.32

RT: retention time; a and b: the slope and the y-intercept of the calibration curve, respectively; *r*^2^: coefficient of determination; LOD: limit of detection; LOQ: limit of quantification; HMF: 5-hydroxymethyl furfural.

**Table 2:** High-performance liquid chromatography method coupled with photodiode array detection (HPLC–PDA) analysis of the main compounds found in carob liqueurs obtained from different extraction methods and after the gastric and intestinal phases of a digestion process.

Liqueur Elaboration	Digestion Process	Concentration (mg/L)		
Method of Extraction	Liqueur	Gallic Acid	HMF	Furfural
Hydro-alcoholic infusion	Undigested	95.25 ± 1.39 ^a^	61.95 ± 0.73 ^a^	17.80 ± 0.07 ^c,d^
	After gastric digestion	93.57 ± 1.38 ^a^	61.30 ± 0.36 ^a^	17.63 ± 0.22 ^c,d^
	After gastrointestinal digestion	86.86 ± 4.37 ^a,b,c^	52.49 ± 1.27 ^b^	14.02 ± 1.65 ^f^
Maceration	Undigested	94.60 ± 3.27 ^a^	58.98 ± 0.57 ^a^	16.96 ± 0.62 ^d,e^
	After gastric digestion	92.05 ± 0.30 ^a,b^	59.20 ± 0.02 ^a^	17.56 ± 0.04 ^c,d^
	After gastrointestinal digestion	82.75 ± 8.06 ^b,c,d^	42.57 ± 0.65 ^d^	11.09 ± 0.05 ^g^
Percolation	Undigested	76.02 ± 9.17 ^b^	47.60 ± 6.13 ^c^	19.25 ± 0.27 ^a,b,c^
	After gastric digestion	68.82 ± 7.31 ^e^	45.46 ± 4.60 ^c,d^	18.26 ± 1.69 ^b,c,d^
	After gastrointestinal digestion	<LOQ	28.33 ± 2.34 ^g^	11.01 ± 1.85 ^g^
	Undigested	74.96 ± 4.67 ^d,e^	33.21 ± 1.33 ^e,f^	12.77 ± 0.98 ^f,g^
Aqueous infusion	After gastric digestion	79.70 ± 4.05 ^c,d^	35.72 ± 0.83 ^e^	17.12 ± 1.24 ^c,d,e^
	After gastrointestinal digestion	<LOD	29.77 ± 0.57 ^f,g^	12.88 ± 0.85 ^f,g^
Distillation	Undigested	n.d.	<LOD	20.86 ± 1.84 ^a^
	After gastric digestion	n.d.	<LOD	20.25 ± 0.58 ^a,b^
	After gastrointestinal digestion	n.d.	<LOD	14.97 ± 0.95 ^e,f^
*Fig spirit*	Undigested	n.d.	n.d.	38.51 ± 0.70

Values are expressed as mean ± standard deviation (*n* = 3). In the same column, values marked with different letters indicate a significant difference (*p* < 0.05). LOQ: limit of quantification; LOD: limit of detection; n.d.: not detected. HMF: 5-hydroxymethyl furfural.

**Table 3:** Correlation matrix (Pearson correlation coefficients) among gallic acid (GA), total phenolic (TPC) and flavonoid (TFC) contents, antioxidant capacity [Trolox equivalent antioxidant capacity (TEAC) and oxygen radical absorbance capacity (ORAC) methods], furfural (F), 5-hydroxymethyl furfural (HMF), and enzyme [acetylcholinesterase (AChE), tyrosinase (Tyr), α-glucosidase (α-gluc) and α-amylase (α-amyl)] inhibitory capacities.

Variables	GA	TPC	TFC	TEAC	ORAC	F	HMF	AchE	Tyr	α-Gluc	α-Amyl
GA	**1**	0.370	−0.296	0.478	0.202	0.031	**0.891**	0.243	**0.603**	0.127	0.114
TPC		1	**0.615**	**0.862**	**0.914**	−0.405	**0.598**	**−0.560**	−0.277	−0.500	0.128
TFC			**1**	0.473	**0.838**	**−0.699**	−0.135	**−0.782**	**−0.829**	−0.443	−0.233
TEAC				**1**	**0.811**	**−0.563**	**0.741**	−0.315	−0.069	−0.169	−0.203
ORAC					**1**	**−0.629**	0.399	**−0.640**	**−0.514**	−0.424	−0.129
F						**1**	−0.088	0.509	0.461	0.053	**0.595**
HMF							**1**	0.203	**0.537**	0.125	0.001
AchE								**1**	**0.809**	**0.833**	−0.198
Tyr									**1**	**0.606**	**−0.559**
α-Gluc										**1**	0.021
α-Amyl											**1**

Bold values indicate that the correlation is significant at the 0.05 level.

## Supplementary Materials

The following are available online at https://www.mdpi.com/2076-3921/8/11/563/s1, Figure S1: HPLC chromatograph of gallic acid (4.75 min), HMF (6.45 min) and furfural (9.65 min) standards, Figure S2: HPLC chromatograph of hydro-alcoholic infusion liqueur; (A) undigested, (B) after gastric digestion and (C) after gastrointestinal digestion, Figure S3: HPLC chromatograph of maceration liqueur; (A) undigested, (B) after gastric digestion and (C) after gastrointestinal digestion, Figure S4: HPLC chromatograph of percolation liqueur; (A) undigested, (B) after gastric digestion and (C) after gastrointestinal digestion, Figure S5: HPLC chromatograph of aqueous infusion liqueur; (A) undigested, (B) after gastric digestion and (C) after gastrointestinal digestion, Figure S6: HPLC chromatograph of distillation liqueur; (A) undigested, (B) after gastric digestion and (C) after gastrointestinal digestion.
