# Supplementary material for: The Influence of In Vitro Gastrointestinal Digestion on the Chemical Composition and Antioxidant and Enzyme Inhibitory Capacities of Carob Liqueurs Obtained with Different Elaboration Techniques
Source: Antioxidants (Basel). 2019 Nov 16;8(11):563. doi: 10.3390/antiox8110563 (PMC6912352; doi:10.3390/antiox8110563)
Supplement: Supplementary file 1 [file antioxidants-08-00563-s001.pdf]

Supplementary information

Influence of *in vitro* gastrointestinal digestion on chemical composition, and antioxidant and enzyme inhibitory capacities of carob liqueurs obtained with different elaboration techniques

Raquel Rodríguez-Solana [1]*, Natacha Coelho [1], Antonio Santos-Rufo [2], Sandra Gonçalves [1], Efrén Pérez-Santín [3] and Anabela Romano [1]*

[1] Faculdade de Ciências e Tecnologia (MeditBio), Universidade do Algarve, Campus de Gambelas, 8005-139 Faro, Portugal; rrsolana@ualg.pt; nrcoelho@ualg.pt; smgoncalves@ualg.pt; aromano@ualg.pt

[2] Laboratory of Plant Pathology, Area of Crop Protection, Andalusian Institute of Agricultural Research and Training (IFAPA), Centro 'Alameda del Obispo', Apartado 3092, Cordoba 14080, Spain; antonio.santos.rufo@juntadeandalucia.es

[3] Universidad Internacional de La Rioja (UNIR), Av. de la Paz, 137, Logroño, 26006 La Rioja, Spain; efren.perez@unir.net

* Correspondence: aromano@ualg.pt; Tel.: +351289800910; +351.289.800900 Ext. 7323 (R.R.S.)

Index

Figure S1. HPLC chromatograph of gallic acid (4.75 min), HMF (6.45 min) and furfural (9.65 min) standards.

Figure S2. HPLC chromatograph of hydro-alcoholic infusion liqueur; **A)** undigested, **B)** after gastric digestion and **C)** after gastrointestinal digestion.

Figure S3. HPLC chromatograph of maceration liqueur; **A)** undigested, **B)** after gastric digestion and **C)** after gastrointestinal digestion.

Figure S4. HPLC chromatograph of percolation liqueur; **A)** undigested, **B)** after gastric digestion and **C)** after gastrointestinal digestion.

Figure S5. HPLC chromatograph of aqueous infusion liqueur; **A)** undigested, **B)** after gastric digestion and **C)** after gastrointestinal digestion.

Figure S6. HPLC chromatograph of distillation liqueur; **A)** undigested, **B)** after gastric digestion and **C)** after gastrointestinal digestion.

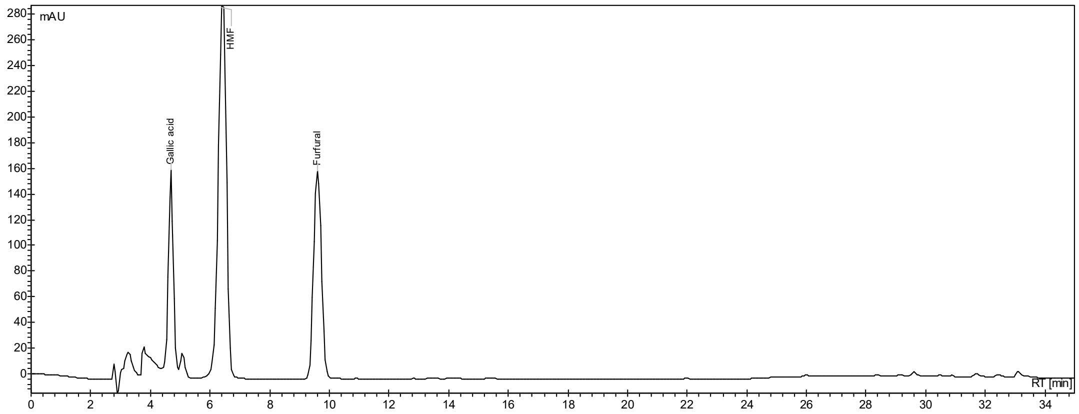

Figure S1. HPLC chromatograph of gallic acid (4.75 min), HMF (6.45 min), and furfural (9.65 min) standards.

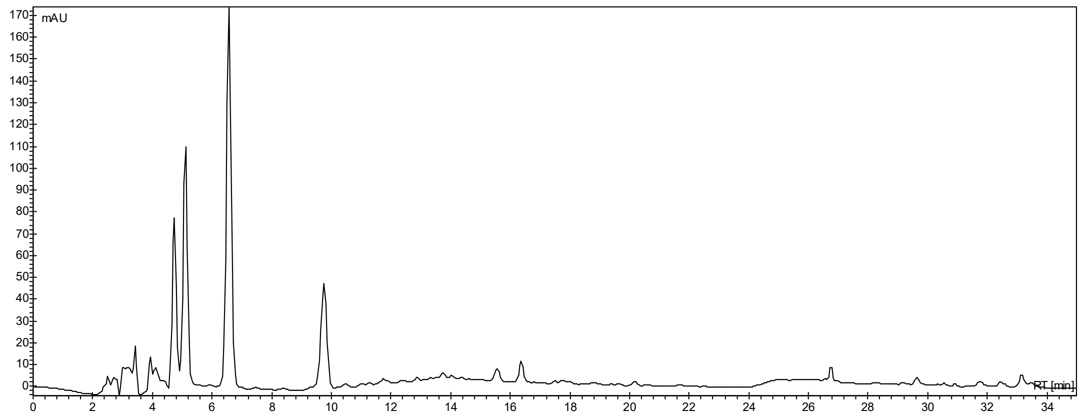

Figure S2A. HPLC chromatograph of hydro-alcoholic infusion liqueur.

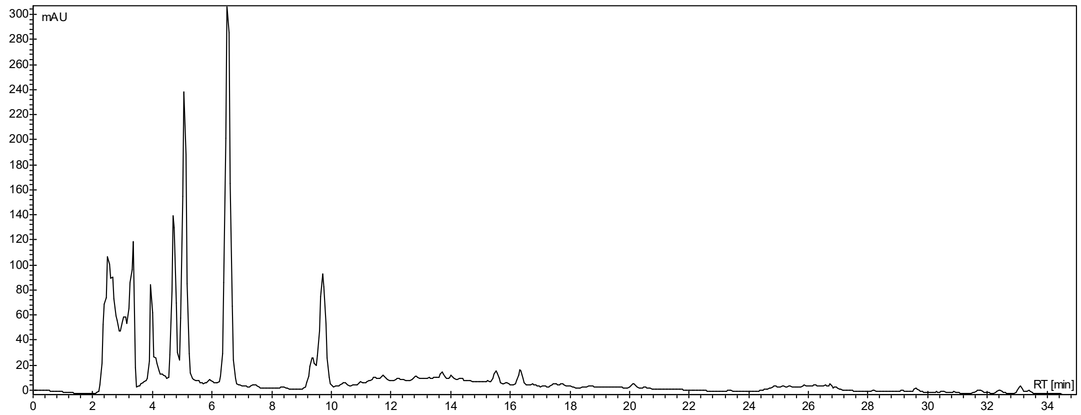

Figure S2B. HPLC chromatograph of hydro-alcoholic infusion liqueur after gastric digestion.

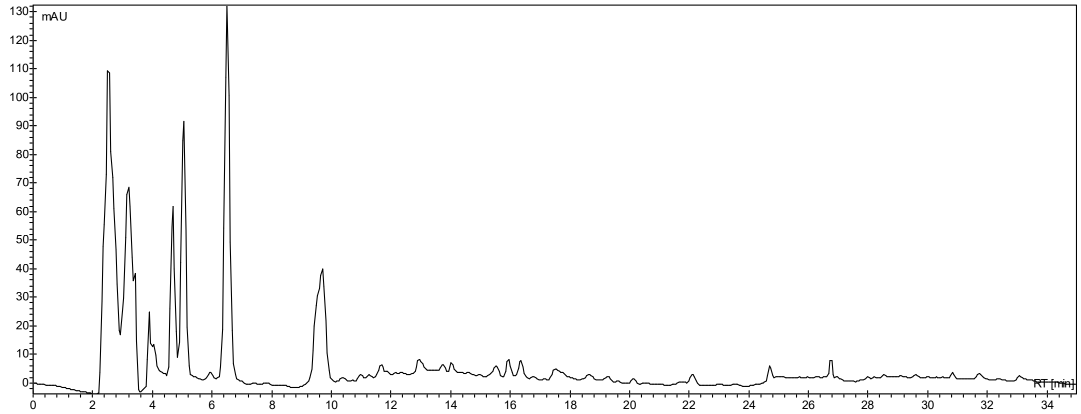

Figure S2C. HPLC chromatograph of hydro-alcoholic infusion liqueur after gastrointestinal digestion.

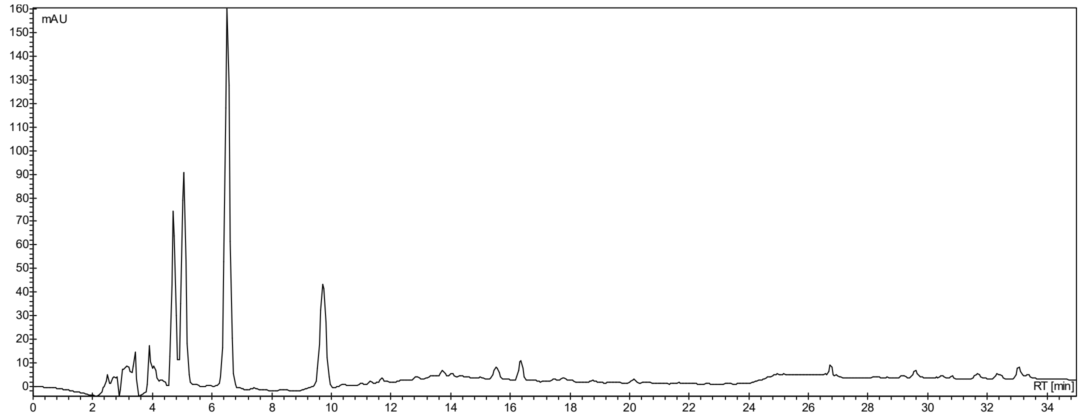

Figure S3A. HPLC chromatograph of maceration liqueur.

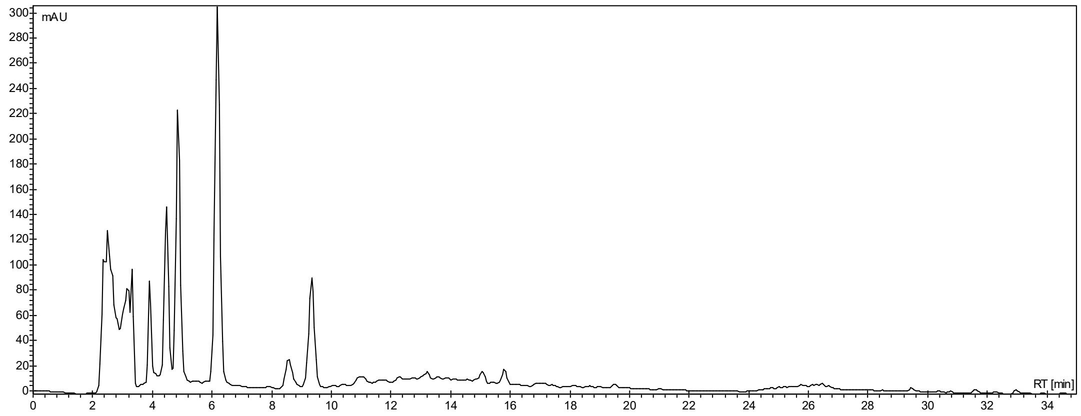

Figure 3B. HPLC chromatograph of maceration liqueur after gastric digestion.

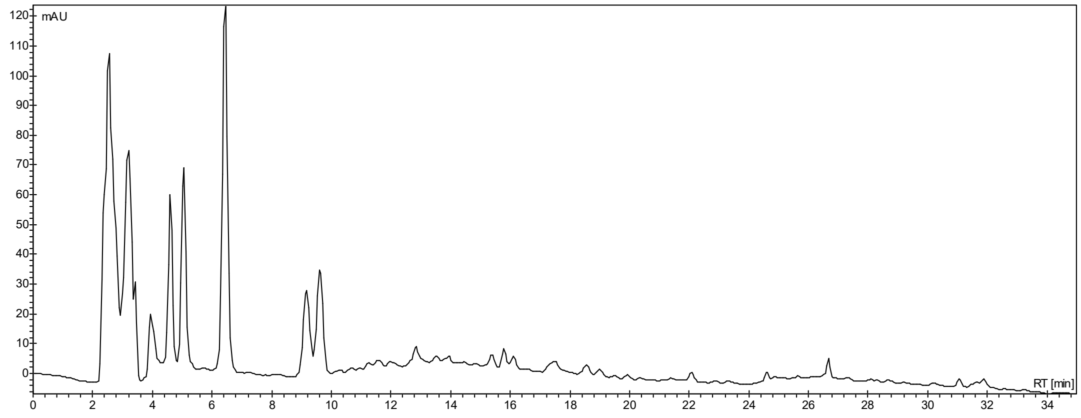

Figure S3C. HPLC chromatograph of maceration liqueur after gastrointestinal digestion.

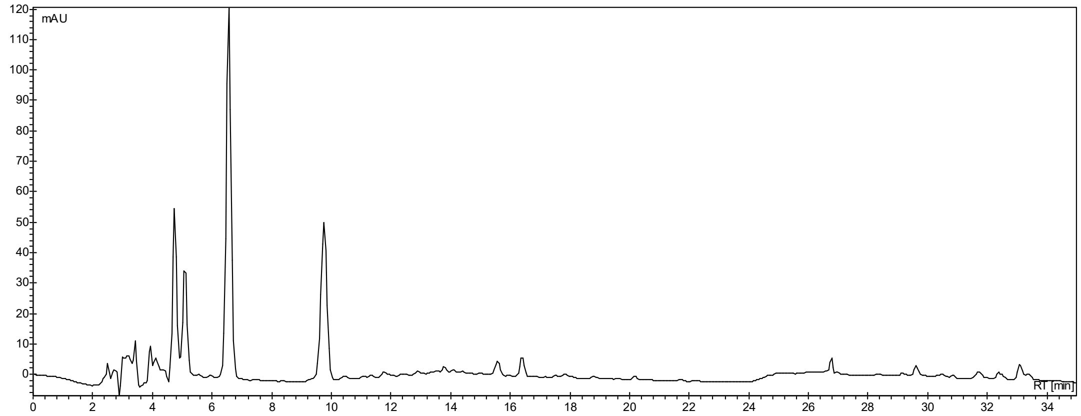

Figure S4A. HPLC chromatograph of percolation liqueur.

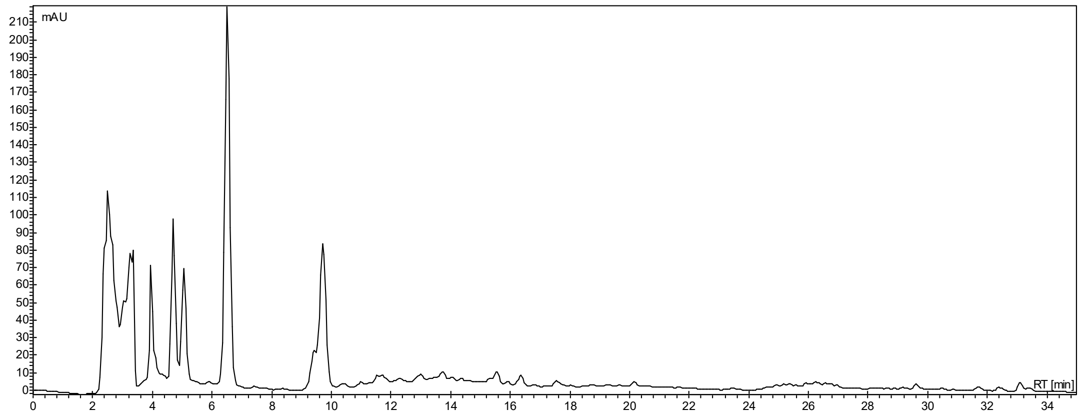

Figure S4B. HPLC chromatograph of percolation liqueur after gastric digestion.

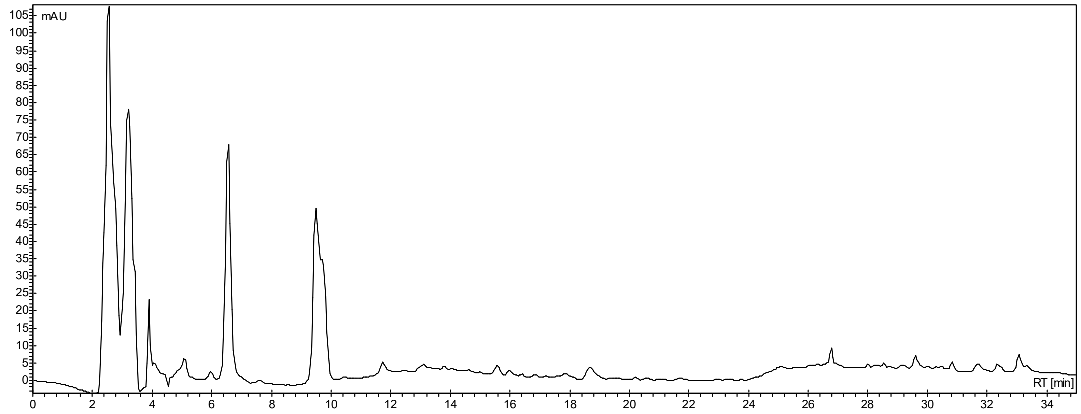

Figure S4C. HPLC chromatograph of percolation liqueur after gastrointestinal digestion.

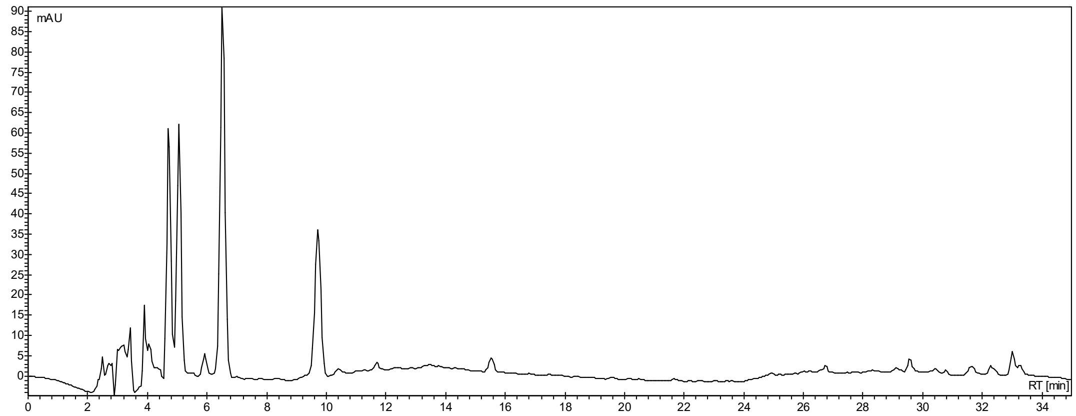

Figure 5A. HPLC chromatograph of aqueous infusion liqueur.

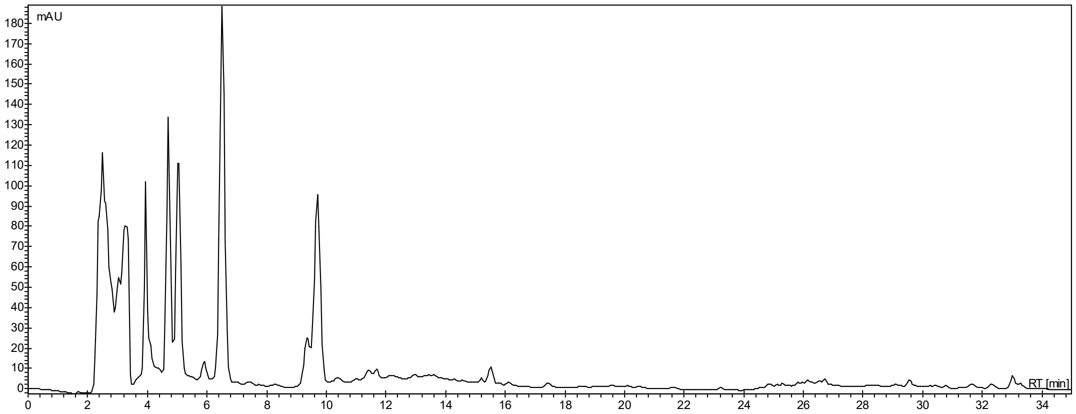

Figure S5B. HPLC chromatograph of aqueous infusion liqueur after gastric digestion.

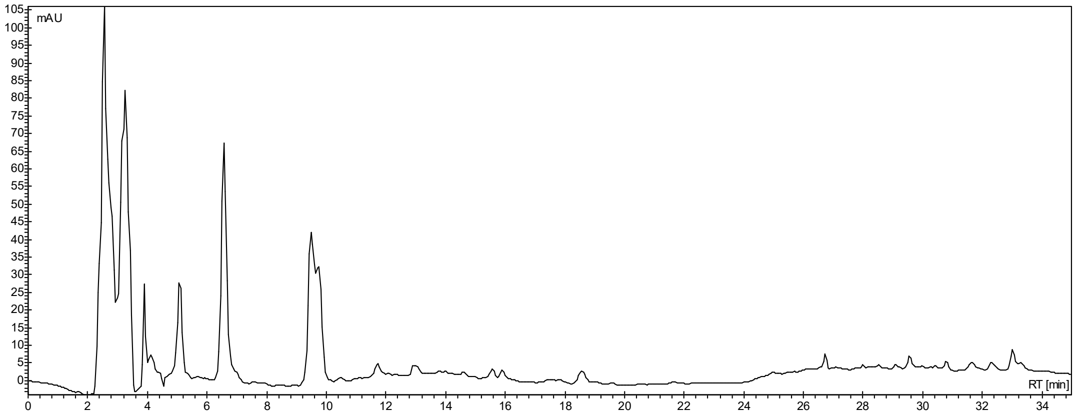

Figure S5C. HPLC chromatograph of aqueous infusion liqueur after gastrointestinal digestion.

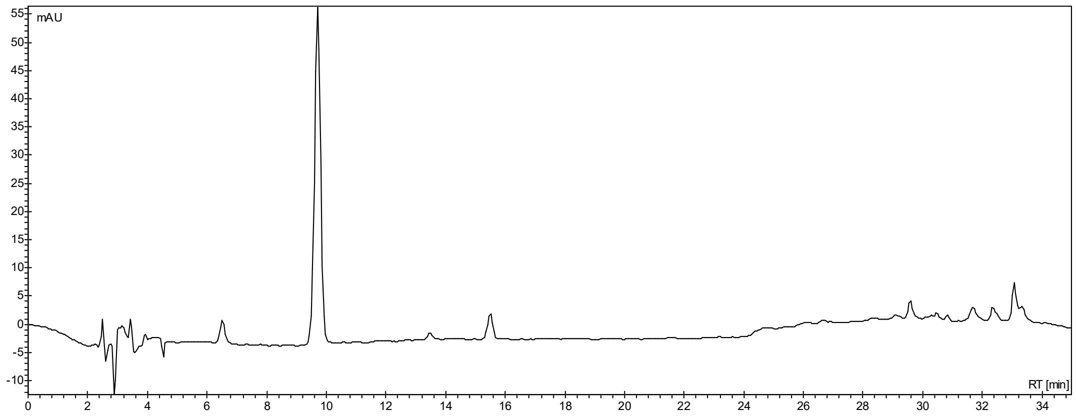

Figure S6A. HPLC chromatograph of distillation liqueur.

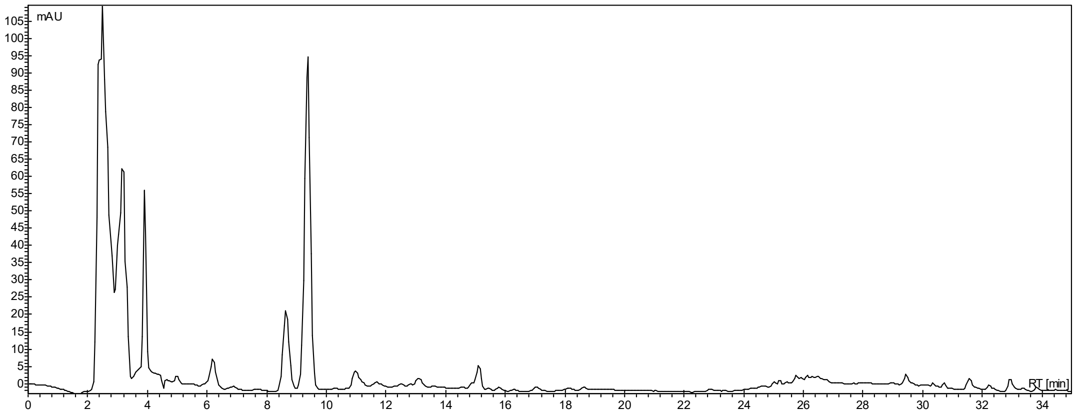

Figure S6B. HPLC chromatograph of distillation liqueur after gastric digestion.

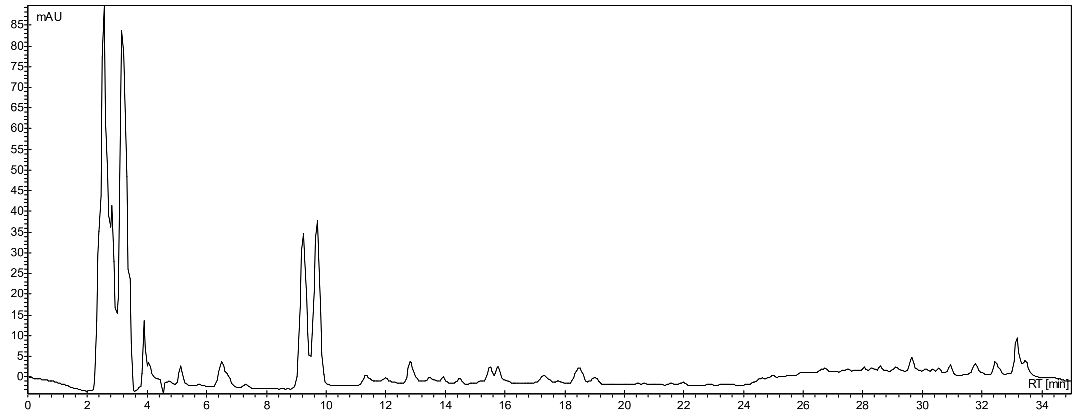

Figure S6C. HPLC chromatograph of distillation liqueur after gastrointestinal digestion.
